# Atypical manifestation of leprosy in a pediatric patient

**DOI:** 10.1590/0037-8682-0278-2024

**Published:** 2025-01-27

**Authors:** Gabriel Castro Tavares

**Affiliations:** 1 Instituto de Puericultura e Pediatria Martagão Gesteira, Serviço de Dermatologia, Rio de Janeiro, RJ, Brasil.

**FIGURE 1:**
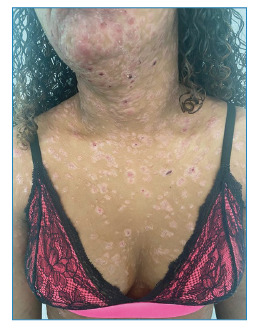
Erythematous and scaly plaques with a crusted center and oval shape located on the anterior chest.

**FIGURE 2:**
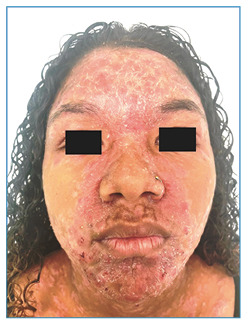
Erythematous-scaly plaques grouped in the frontal and central-facial region.

**FIGURE 3:**
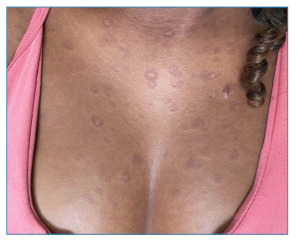
Erythematous brownish to violet plaques, non-scaling, with infiltrated edges and annular shape located on the anterior chest.

A teenager, 14 years old, reported the progressive appearance of skin lesions for two months, which was unassociated with other symptoms. Physical examination revealed erythematous and scaly plaques, some with a crusted center and oval shape, with a diffuse and symmetrical distribution located on the trunk, upper limbs, and face, grouped in the frontal and center-facial regions (Figures 1 and 2). There was no change in the peripheral nerves. An incisional biopsy was performed on a trunk lesion compatible with reactional leprosy. A diagnosis of dimorphic leprosy associated with a reverse reaction was reached after clinical-pathological correlation. The treatment consisted of oral prednisone (1 mg/kg/day) combined with rifampicin, clofazimine, and dapsone for 12 months until clinical improvement of the reaction was observed. The clinical appearance of the skin lesions changed after one week of prednisone treatment. Physical examination revealed erythematous brownish-to-violet plaques, non-scaling, with infiltrated edges and an annular shape (Figure 3), compatible with the classic picture of leprosy after improvement in the reaction. Although leprosy is an endemic disease in Brazil, it remains underdiagnosed. It is caused by *Mycobacterium leprae* and primarily affects the skin and peripheral nerves, causing deformities and functional disabilities if not treated early. Although the diagnosis is based on clinical findings, the inflammatory nature of the leprosy reaction modifies the classic presentation of the disease^1^ ^-^ ^3^, so a histopathological examination is essential to confirm the diagnosis and initiate treatment early.
